# Amyloid β Levels in Human Red Blood Cells

**DOI:** 10.1371/journal.pone.0049620

**Published:** 2012-11-15

**Authors:** Takehiro Kiko, Kiyotaka Nakagawa, Akira Satoh, Tsuyoshi Tsuduki, Katsutoshi Furukawa, Hiroyuki Arai, Teruo Miyazawa

**Affiliations:** 1 Food and Biodynamic Chemistry Laboratory, Graduate School of Agricultural Science, Tohoku University, Sendai, Japan; 2 Life Science Institute, Yamaha Motor Company, Ltd., Shizuoka, Japan; 3 Laboratory of Food and Biomolecular Science, Graduate School of Agricultural Science, Tohoku University, Sendai, Japan; 4 Department of Geriatrics and Gerontology, Institute of Development, Aging and Cancer, Tohoku University, Sendai, Japan; Hirosaki University Graduate School of Medicine, Japan

## Abstract

Amyloid β-peptide (Aβ) is hypothesized to play a key role by oxidatively impairing the capacity of red blood cells (RBCs) to deliver oxygen to the brain. These processes are implicated in the pathogenesis of Alzheimer's disease (AD). Although plasma Aβ has been investigated thoroughly, the presence and distribution of Aβ in human RBCs are still unclear. In this study, we quantitated Aβ40 and Aβ42 in human RBCs with ELISA assays, and provided evidence that significant amounts of Aβ could be detected in RBCs and that the RBC Aβ levels increased with aging. The RBC Aβ levels increased with aging. On the other hand, providing an antioxidant supplement (astaxanthin, a polar carotenoid) to humans was found to decrease RBC Aβ as well as oxidative stress marker levels. These results suggest that plasma Aβ40 and Aβ42 bind to RBCs (possibly with aging), implying a pathogenic role of RBC Aβ. Moreover, the data indicate that RBC Aβ40 and Aβ42 may constitute biomarkers of AD. As a preventive strategy, therapeutic application of astaxanthin as an Aβ-lowering agent in RBCs could be considered as a possible anti-dementia agent.

**Trial Registration:**

Controlled-Trials.com ISRCTN42483402

## Introduction

Alzheimer's disease (AD) is the most common form of dementia. Since AD is associated with the progressive accumulation of amyloid β-peptide (Aβ) in the human brain, a pathogenic role of Aβ in the brain has been widely recognized [1], [2].

Over the last decade or so, the presence of Amyloid β-peptide (Aβ) in peripheral blood plasma has received increasing attention [3]–[6], and plasma Aβ is hypothesized to readily contact red blood cells (RBCs) and impair the capacity of RBCs in circulating human blood [7], [8]. Our group and other researchers have investigated the hypothesis, and found that Aβ induces oxidative injury to RBC by binding to them, and causing accumulation of phospholipid hydroperoxides (PLOOH), a specific marker for RBC membrane oxidative injury [9], [10]. Aβ also induces the binding of erythrocytes to endothelial cells and decreases endothelial viability, perhaps by the generation of oxidative and inflammatory stress [11]. These studies [9]–[11] provide a possibility that Aβ plays a key role in blood and oxidatively impairs RBC function (e.g., oxygen delivery to the brain), thereby potentially facilitating AD. However, to the best of our knowledge, no extensive study of the presence and distribution of Aβ in human RBC has been undertaken.

The aim of this study was to ascertain the distribution of Aβ in the RBCs of young and senior subjects by applying a commercial ELISA assay. The RBC Aβ concentrations were compared between young and senior subjects and also compared to plasma Aβ levels. In addition, we previously conducted a randomized, double-blind, placebo-controlled human study to evaluate whether nutritional supplementation with the antioxidant astaxanthin (a polar carotenoid) affected RBC PLOOH [12]. Thus, RBCs that had been obtained from the human study [12] were subjected to Aβ determination in order to evaluate the relationship between RBC Aβ and the antioxidant/oxidant profile.

## Materials and Methods

### Ethics Statement

The study was conducted in accordance with the Declaration of Helsinki and approved by the Ethics Committee of the Anti-Aging Science (Tokyo, Japan; ethics No. I030807). All of the subjects provided written informed consent to the experimental protocol before participating in the study.

### Blood Samples from Young and Senior Volunteers

Twenty-four young healthy human volunteers [12 men and 12 women, between 22 and 29 years of age (mean ± SE, 24.2±0.6)] and 38 senior healthy volunteers [20 men and 18 women, between 48 and 69 years of age (mean ± SE, 56.2±1.0)] participated in this study. Blood was collected into a tube containing EDTA-2Na as an anticoagulant and was subjected to centrifugation at 1,000 *g* for ten min at 4°C. After the plasma and buffy coat were removed, RBCs were washed three times with phosphate buffered saline (PBS, pH 7.4) to prepare packed cells.

### Measurement of Aβ40 and Aβ42 in RBCs and Plasma

For determination of Aβ40 and Aβ42 in RBCs, human β Amyloid (1–40) ELISA kits (WAKO, Osaka, Japan) and human β Amyloid (1–42) ELISA kits (WAKO) were used, respectively. These kits are commercially available and used worldwide. We tested conditions for measurement of RBC Aβ, and the optimized protocol is as follows. Packed cells (200 µL) were mixed with 200 µL of water and one mL of 70% formic acid. A 40 µL aliquot was collected and mixed with 760 µL of 1 mol/L Tris-HCl with protease inhibitors, and the mixture was diluted two-fold with the standard diluent present in each Aβ40 and Aβ42 ELISA kit. A 100 µL aliquot was subjected to either the Aβ40 or the Aβ42 ELISA kit (in triplicate). Briefly, aliquots (100 µL) were transferred to BAN50-coated (specific for the N-terminal portion of human Aβ1-16) 96 well microplates and incubated overnight at 4°C. After washing five times with the kit’s wash solution, HRP-conjugated BA27 (specific for the C-terminal portion of human Aβ40) or HRP-conjugated BC05 (specific for the C-terminal portion of human Aβ42) was added and incubated at 4°C for one h. After washing in the same manner, TMB solution from the ELISA kit was added and incubated for 30 min. Stop solution in each Aβ40 and Aβ42 ELISA kit was added and absorbance was read at 450 nm using a microplate reader (GENios, TECAN Co., Ltd.). For plasma Aβ40 and Aβ42, these were determined according to the ELISA kit protocol.

### Previous Human Study Protocol

As described above, we previously conducted a randomized, double-blind, placebo controlled human study to evaluate whether antioxidant supplementation (astaxanthin) affected RBC phospholipid peroxidation [12]. In this study, blood samples (RBC and plasma) that had been obtained from the human study [12] were subjected to Aβ determination. That previous human study protocol was organized as follows. A total of 30 healthy subjects (15 men and 15 women), between 50 and 69 years of age (mean ± SE, 56.3±1.0), randomly received zero mg (placebo), six mg, or 12 mg antioxidant (astaxanthin, Puresta®, Yamaha Motor Co., Ltd.; Shizuoka, Japan). During the 12-week trial, subjects ingested one of the three astaxanthin doses (zero, six, or 12 mg) capsules with an appropriate amount of water once daily after breakfast. Before and after the supplementation period (weeks zero and 12), blood samples were collected from the subjects. From the blood samples, RBC and plasma were prepared, and their PLOOH and antioxidants (carotenoids and tocopherols) were measured by HPLC techniques [9], [12]–[16].

### Statistical Analyses

Data are presented as means ± SE. Differences in Aβ concentrations between young and senior subjects were compared using Student’s *t*-test or Welch’s *t*-test for equal or unequal variances; the Mann-Whitney *U* test was used when the distribution was skewed. For correlation analysis, Pearson’s correlation coefficient test for normal data or Spearman’s rank correlation coefficient test for nonparametric data was used. A difference was considered significant at *P*<0.05.

## Results

### RBC Aβ in Young Human Volunteers

The ELISA assay kit is designed to be used for the quantitative determination of Aβ40 and Aβ42 in human fluid samples including plasma, CSF, tissue homogenate, and tissue culture media [3], [17]–[19]. The ELISA kit was developed by Suzuki et al. and shows extremely highly sensitivity and reproducibility [20]. Using the kit, we found that Aβ could be detected in RBC packed cells. Therefore, conditions for the measurement of RBC Aβ40 and Aβ42 were optimized, and Aβ40 and Aβ42 levels in RBCs of young healthy volunteers were determined. As a result, RBC Aβ concentrations were calculated to be 5.32±0.21 pmol/g hemoglobin for Aβ40 and 2.09±0.06 pmol/g hemoglobin for Aβ42 (Table 1). If Aβ levels in RBC were compared to plasma, Aβ40 and Aβ42 levels in RBC were about 8 and 14 times higher than those of plasma, respectively. The experiments also confirmed that the RBC Aβ42/Aβ40 ratio was about 1.8 times higher than plasma (Table 1).

**Table 1:** Amyloid β levels in RBC and plasma of young healthy human volunteers and senior subjects.

Parameters	Young healthy human volunteers	Senior healthy human volunteers
Total number of subjects	24	38
Males	12	20
Females	12	18
Age (years)	24.2±0.6	56.2±0.9
RBC Aβ40 (pmol/g hemoglobin)	5.33±0.21	8.16±0.47*
RBC Aβ42 (pmol/g hemoglobin)	2.09±0.06	3.81±0.22*
RBC Aβ42/40	0.41±0.03	0.51±0.03*
PLOOH (pmol/mL packed cells)	8.4±0.7	15.8±1.2*
Plasma Aβ40 (pmol/g protein)	0.669±0.032	0.806±0.029*
Plasma Aβ42 (pmol/g protein)	0.147±0.006	0.242±0.019*
Plasma Aβ42/40	0.232±0.013	0.298±0.018

Means ± SE are shown.

Significantly different between young healthy volunteers and senior subjects: *P<0.01.

### Comparison of RBC Aβ Concentrations between Young and Senior Subjects

**Figure 1:**
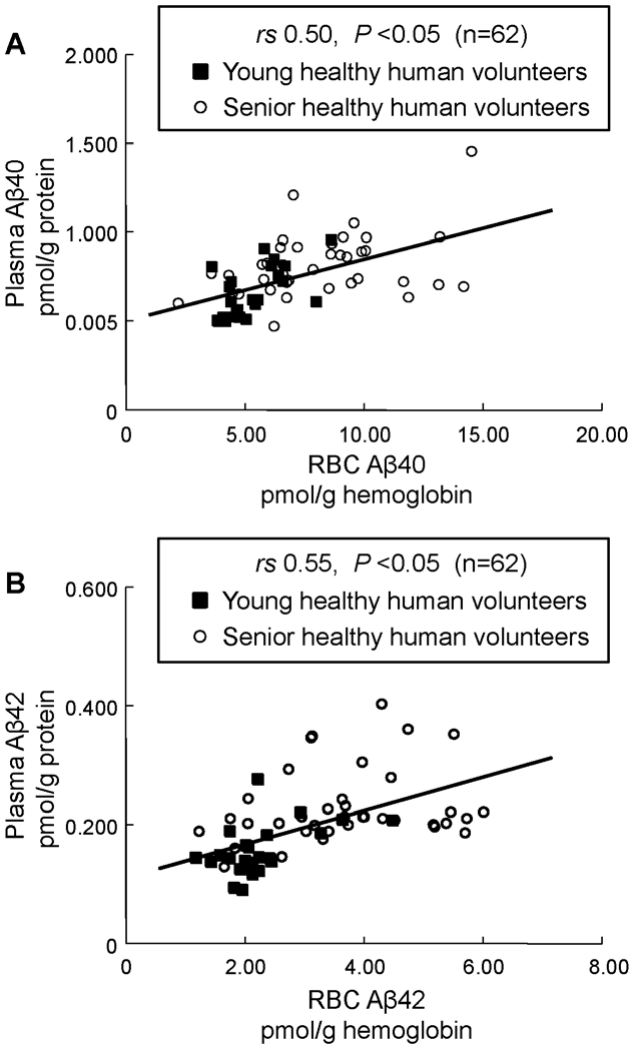
Correlation between RBC and plasma Aβ40 (A) or Aβ42 (B) concentrations of young healthy human volunteers and senior subjects (N = 62). X-axis is the concentration of RBC Aβ. Y-axis is concentration of plasma Aβ.

It was found that the concentrations of RBC Aβ40 and Aβ42 in senior healthy volunteers were significantly higher than those of young healthy volunteers (Table 1). The plasma Aβ40 and Aβ42 in elderly subjects were also higher than those of young volunteers. When we analyzed the relationship between Aβ in RBC and plasma, significant positive correlations were found (Fig. 1). On the other hand, RBC PLOOH levels in senior subjects were also higher than those of young volunteers.

### Astaxanthin Antioxidant Supplementation Trial: RBC Aβ and the PLOOH Profile

**Figure 2:**
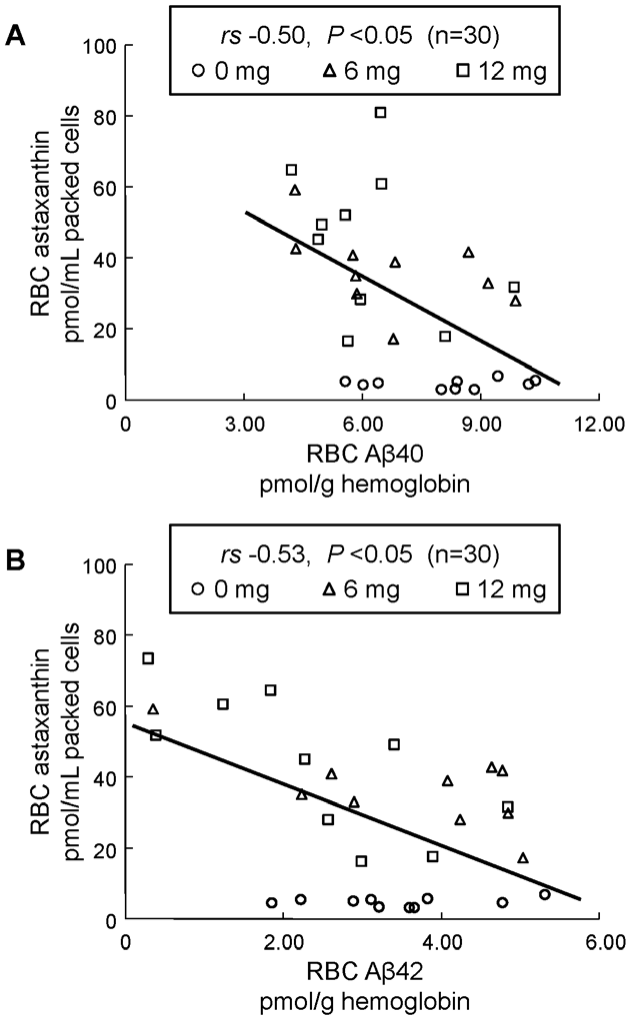
Correlation between RBC astaxanthin and Aβ40 (A) or Aβ42 (B) concentrations after 12 weeks administration of astaxanthin (N = 30). X-axis is the concentration of RBC Aβ. Y-axis is concentration of RBC astaxanthin that had been measured in our former human study [12].

**Figure 3:**
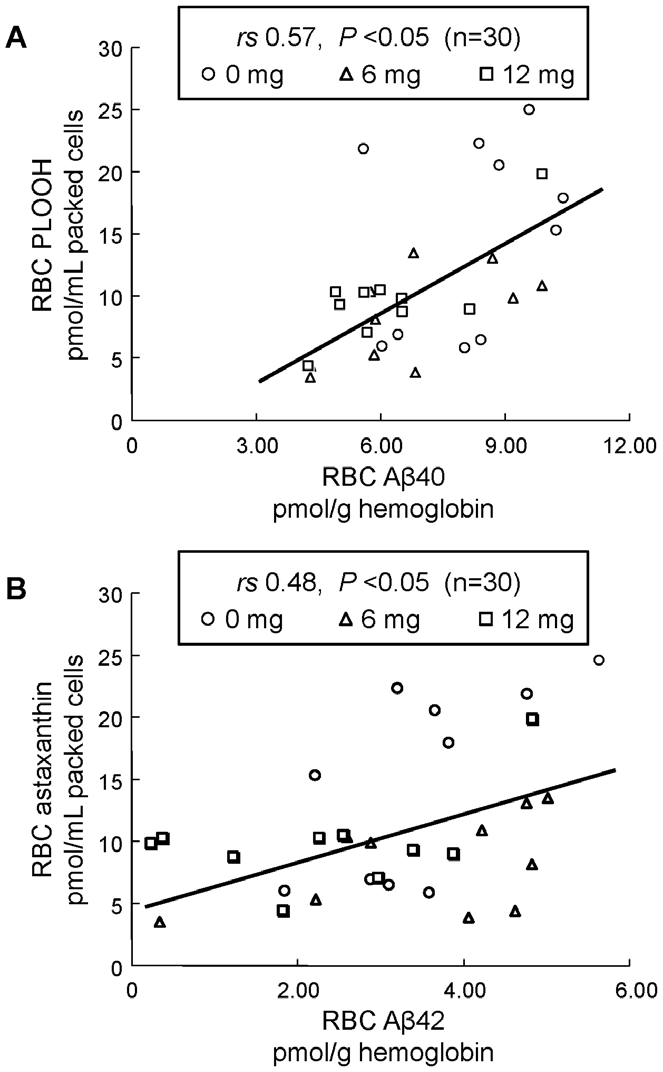
Correlation between RBC PLOOH and Aβ40 (A) or Aβ42 (B) concentration after 12 weeks administration of astaxanthin (N = 30). X-axis is concentration of RBC Aβ. Y-axis is concentration of RBC PLOOH [phosphatidylcholine hydroperoxide (PCOOH) and phosphatidylethanolamine hydroperoxide (PEOOH)] that had been measured in our former human study [12].

As already reported in our former study [12], after human volunteers (senior subjects) received supplementation with the antioxidant astaxanthin, RBC astaxanthin concentrations were significantly increased and the RBC PLOOH concentration decreased. For other parameters, astaxanthin supplementation showed a safety profile with no side effects (e.g., death of RBC, leukocytosis, and inflammatory conditions) [12]. In the present study, we measured RBC Aβ40 and Aβ42 concentrations, and these levels were found to be significantly decreased after supplementation (Fig. 1A, Table 2). In addition, there were inverse relationships between RBC Aβ and astaxanthin concentrations (Fig. 2). On the other hand, astaxanthin supplementation did not affect the levels of plasma Aβ40 and Aβ42 (Fig. 1B, Table 2). When we analyzed the relationship between RBC Aβ and an oxidative stress marker (PLOOH), we found a significant positive correlation between RBC PLOOH and Aβ concentrations (Fig. 3).

**Table 2:** Changes in Amyloid β levels in RBC and plasma before and after a 12 week administration of 0, 6 or 12 mg astaxanthin.

Parameters	0 mg	6 mg	12 mg
Age	56.6±1.4	56.3±2.1	56.1±1.6
Total number of subjects	10	10	10
Males	5	5	5
Females	5	5	5
**RBC Aβ40 (pmol/g hemoglobin)**			
Before administration	7.89±0.46	8.13±0.65	8.36±0.55
After administration	8.23±0.54	7.08±0.56	6.31±0.53*
**RBC Aβ42 (pmol/g hemoglobin)**			
Before administration	3.90±0.29	4.24±0.37	4.08±0.55
After administration	3.69±0.28	3.60±0.34	2.40±0.47*
**Plasma Aβ40 (pmol/g protein)**			
Before administration	0.797±0.050	0.817±0.051	0.802±0.034
After administration	0.804±0.040	0.786±0.052	0.747±0.041
**Plasma Aβ42 (pmol/g protein)**			
Before administration	0.223±0.011	0.236±0.031	0.266±0.020
After administration	0.232±0.021	0.204±0.029	0.243±0.021

Means ± SE are shown.

Significantly different between before and after astaxanthin administration: *P<0.05.

Blood samples (RBC and plasma) that had been obtained from our former human study [12] were subjected to Aβ determination.

## Discussion

The brain is generally regarded as the origin of the Aβ that is deposited in plaques of AD patients [5], [21]. Plasma Aβ and CSF concentrations are believed to be in a dynamic equilibrium [22], [23], suggesting that increased Aβ production in the brain could be associated with increased Aβ concentrations in blood plasma. This means that brain Aβ is transferred across the blood-brain barrier to the plasma [24], [25]. As proof of the transfer, peripheral administration of anti-Aβ antibody (m266) to PDAPP transgenic mice (AD-model mice) increased plasma Aβ up to 1000-fold [26]. Additional evidence for the presence of Aβ in blood plasma was obtained in studies of platelets [27]. As mentioned in the Introduction, our group and other researchers found that Aβ is capable of binding to RBC *in vitro* as well as *in vivo* animal studies [9]. Thus, it is likely that Aβ in peripheral blood plasma may readily contact RBC in circulating human blood.

Although plasma Aβ has been investigated thoroughly in previous studies [1], [5], [21], [28], little attention has been paid to RBC Aβ. In the present study, we provide evidence that Aβ is indeed present in human RBC. We found that RBC Aβ40 and Aβ42 levels in healthy human volunteers were about 8- and 14-times higher than plasma Aβ40 and Aβ42, respectively (Table 1), when RBC Aβ levels per g hemoglobin were compared to plasma Aβ levels per g protein. These results suggested that plasma Aβ42 and Aβ40 readily bind to RBC, and this may provide an explanation for lower concentrations of “unbound” Aβ42 and Aβ40 in human plasma [3]. In addition, the experiments confirmed that the RBC Aβ42/Aβ40 ratio was about 1.8-times higher than in plasma (Table 1). This may be related to the fact that Aβ42 interacts with RBC more avidly than Aβ40 [9], [29], probably because two additional hydrophobic amino acids at the C-terminus of Aβ42 increase the rate of Aβ insertion into the RBC bilayer [30].

We also found that RBC Aβ40 and Aβ42 levels in healthy elderly subjects were higher than in young volunteers. It was reported that brain as well as plasma Aβ (Aβ40 and Aβ42) levels tend to increase according to age [31]. The age-related increase in plasma Aβ could be connected to increases in Aβ production or a reduction in Aβ clearance in the brain. These shifts may be related to changes in the central or peripheral activity of Aβ synthetic enzymes (e.g., β-secretase or γ-secretase) or Aβ catabolic enzymes (e.g., insulin-degrading enzyme or neprilysin) associated with aging. Therefore, the age-related enhanced binding of Aβ to RBC may reflect age-dependent changes in Aβ metabolism. Since significant positive correlations were observed between RBC and plasma Aβ concentrations (Fig. 1), this may strengthen the hypothesis.

In order to supply oxygen to the brain, RBCs must deform as they pass through the narrow pores of capillaries. However, RBC deformability reportedly decreases when Aβ adheres to RBC [32]. Thus, the interaction of Aβ with RBC may decrease blood flow, impair oxygen delivery to the brain and contribute to brain hypoxia [32]. These processes are implicated in the pathogenesis of AD. In support of these notions, a relationship between blood (plasma) Aβ and AD was observed in Down syndrome patients, among whom those with elevated Aβ levels in plasma were reported to have a greater risk of developing AD [33]. Additional research (e.g., measurement of Aβ levels in RBCs of AD patients) would be necessary to confirm these hypotheses.

On the other hand, as a preventive strategy, compounds that are capable of minimizing the accumulation of Aβ in blood might be useful therapeutically. In this study, we showed that after astaxanthin supplementation, Aβ40 and Aβ42 concentrations in RBC (but not plasma) were significantly decreased (Fig. 1, Table 2). In addition, inverse relationships between RBC Aβ and astaxanthin levels were found (Fig. 2). Our previous *in vitro* and *in vivo* murine studies also indicated that carotenoid supplementation, especially astaxanthin, could attenuate Aβ-induced oxidative stress in RBCs [9]. It is therefore likely that carotenoids (astaxanthin) act as antioxidants and/or reduce the binding of Aβ to RBCs, thereby improving the resistance of RBCs to Aβ-induced oxidative damage. For other carotenoid, β-carotene reportedly inhibited fibrillation and oligomerization of Aβ [34], [35], indicating a possibility that carotenoid moieties may bind to C-terminal portion of Aβ, thereby inhibiting the binding of Aβ to RBC. On the other hand, for currently unknown reasons, astaxanthin changed the levels of Aβ in RBC but not in plasma. This may be related to Aβ clearance from plasma, since excessive plasma Aβ is reportedly cleared from the circulation by mainly hepatic Aβ uptake through the interactions with liver low-density lipoprotein receptor-related protein (LRP-1) [36]–[38]. Further studies are needed to evaluate the effectiveness and mechanisms by which carotenoid (astaxanthin) could be beneficial for the treatment of dementia.

Studies have reported that Aβ elicits neurotoxic activity via generation of reactive oxygen species (ROS) [39]. The mechanism by which Aβ generates ROS is not fully understood, although one study implicates involvement of the methionine residue at position 35 of Aβ [40]. If, indeed, Aβ induces ROS, it could in turn trigger membrane oxidative injury in RBCs. Because Aβ seems to cause RBC aggregation and hemolysis [9], it is plausible that Aβ-induced hemolysis enhances a cascade of oxidative reactions in RBC. These reactions produce superoxide, which dismutates to form hydrogen peroxide. These ROS cause formation and accumulation of RBC PLOOH, and this could increase membrane rigidity and decrease the deformability of RBCs. In concordance with these considerations, positive correlations between RBC Aβ and PLOOH were found in the present study (Fig. 3).

In conclusion, we provided evidence that Aβ40 and Aβ42 concentrations were much higher in RBCs than in plasma and that RBC Aβ levels increased with aging. We also found that after astaxanthin supplementation, there was a decrease in RBC Aβ concentrations. The RBC Aβ levels were positively correlated with RBC PLOOH, and inversely correlated with RBC astaxanthin. Based on the present findings, we are currently collecting the blood samples from living AD subjects to investigate the pathogenic roles of RBC Aβ and usability of RBC Aβ as biomarkers of AD. Also, we are investigating therapeutic application of carotenoids (astaxanthin) for possible anti-dementia agents. These results will be presented in the near future as a different study.
